# Optimising adolescents and young adults’ utilisation of sexual and reproductive health and HIV services in Chad: a sensemaking approach

**DOI:** 10.1136/bmjgh-2024-017763

**Published:** 2025-03-26

**Authors:** Esias Bedingar, Ferdinan Paningar, Ngarossorang Bedingar, Eric Mbaidoum, Naortangar Ngaradoum, Rifat Atun, Aisha Yousafzai

**Affiliations:** 1 Global Health and Population, Harvard University T H Chan School of Public Health, Boston, Massachusetts, USA; 2 Alma, Centre de Recherche en Systèmes de Santé, N’Djamena, Chad; 3 Bucofore, N’Djamena, Chad; 4 Blue Cross Chad, N’Djamena, Chad; 5 Réseau National des Personnes Vivants avec le VIH, N’Djamena, Chad; 6 School of Public Health, Harvard University, Boston, Massachusetts, USA

**Keywords:** HIV, Health policy, Health services research, Health systems

## Abstract

**Background:**

Adolescents and young adults (AYA) aged 15–24 years account for nearly half of all new HIV infections globally, with over 80% residing in sub-Saharan Africa, where AIDS is a leading cause of death. In Chad, HIV/AIDS significantly impacts AYA, despite a reduction in national prevalence from 1.6% in 2010 to 1.0% in 2022. AYA still contribute to 26.3% of new infections, with young women facing four times the risk compared with young men. Barriers throughout the care continuum, including transitions from paediatric to adult services, contribute to an overall antiretroviral therapy (ART) coverage of 47% in 2023, with only 27% of males compared with 58% of females receiving treatment. These challenges impact access to sexual and reproductive health services and effective help-seeking.

**Methods:**

This study used participatory action research with a sensemaking theoretical lens. From December 2021 to March 2024, data were collected from 52 AYA (aged 15–24, both HIV-positive and HIV-negative) in focus group discussions. Data were analysed using a modified grounded theory approach, with coding and category development guided by the constant comparison method.

**Results:**

Findings indicate that empowering AYA in health decision-making requires an adaptable approach considering individual experiences, interpersonal influences and systemic factors. Such an approach can enhance engagement with healthcare services, increase service utilisation and treatment adherence and inform policy redesign for more effective service delivery.

**Conclusion:**

This study advances the understanding of sensemaking in healthcare, illustrating how AYA in Chad navigate and interpret the healthcare system. It highlights the need for AYA-friendly services that foster clear communication, trust and responsive care. Strengthening health systems through adolescent-responsive policies, workforce training and decentralised service delivery can enhance AYA engagement, service utilisation and retention in care. Aligning services with AYA perspectives through community-driven approaches and system reforms can improve healthcare accessibility and outcomes in similar contexts.

**WHAT IS ALREADY KNOWN ON THIS TOPIC**

- Adolescents and young adults (AYA) aged 15–24 contribute to nearly half of all new HIV infections globally, with over 80% of AYA living with HIV residing in sub-Saharan Africa, where AIDS remains a leading cause of AYA mortality.
- Adolescence is a critical period marked in sexual and reproductive health (SRH) and HIV contexts. Despite intervention efforts, significant barriers—including psychological, financial and systemic—persist in preventing effective healthcare engagement among AYA.

**WHAT THIS STUDY ADDS**

- This study introduces an adaptive sensemaking framework to better understand how AYA navigate SRH and HIV care in Chad, revealing that individual health decisions are deeply shaped by personal experiences, interpersonal influences and systemic factors.
- It highlights the role of collective sensemaking through social networks and peer interactions, emphasising the need for healthcare environments that are not physically accessible but also culturally sensitive and responsive to AYA-specific needs.

**HOW THIS STUDY MIGHT AFFECT RESEARCH, PRACTICE OR POLICY**

- The findings suggest that integrating an AYA-centred design into SRH and HIV services, which addresses psychological, financial and logistical barriers, can significantly improve AYA engagement and adherence to care.
- This study informs policy by advocating for community-driven participatory approaches and the incorporation of sensemaking theories to redesign AYA-friendly health services that are inclusive, empowering and contextually relevant.

## Introduction

 Adolescents and young adults (AYA) aged 15–24 years contribute to almost half of all new HIV infections globally, with more than 80% of these AYA living in sub-Saharan Africa (SSA), where AIDS is a leading cause of AYA mortality.^1^,^6^ According to the 2023 UNAIDS Global AIDS Update, global HIV prevalence has shown a declining trend due to scaled-up prevention and treatment efforts.^7^ However, significant gender disparities persist. In SSA, adolescent girls and young women (AGYW) account for 63% of all new HIV infections among AYA,^8^ driven by social and structural inequalities, restricted educational opportunities and pervasive gender-based violence.^9 10^ In Chad, the HIV epidemic reflects these trends. AYA remain disproportionately affected, accounting for one-third of new infections,^7^ especially AGYW with HIV rates four times higher than those of young men due to factors such as early marriage, economic dependence and limited access to sexual and reproductive health (SRH) education and services.^10^,^12^

Chad is a low-income, fragile country with a healthcare system that faces chronic underfunding, limited infrastructure and a severe shortage of healthcare personnel.^12^ These systemic weaknesses pose significant barriers to AYA seeking SRH and HIV services.^12^ Access is further constrained by fragmented service delivery, geographical disparities and a lack of adolescent-friendly healthcare settings.^12^ Stigma and discrimination, both at the community level and within healthcare facilities, further deter AYA from accessing essential services.^13^ The societal stigma surrounding HIV, coupled with fear of judgement or disclosure of status, further limits AYA engagement with the healthcare system.^13 14^ Even where services exist, their physical accessibility and quality are often inconsistent, particularly in rural areas.^13^,^16^

Although school-based and community programmes, like the ‘Life Skills and Peer Education’ initiative by a Chadian non-governmental organisation, called Blue Cross Chad, aim to enhance AYA knowledge and prevention practices, they often fall short of comprehensively addressing the needs of all AYA.^17^,^19^ Furthermore, while peer-education programmes in schools have shown success in fostering trust and facilitating SRH and HIV discussions,^1314 17^,^19^ AYA not part of these programmes frequently rely on informal networks and social media for information, which can be inconsistent and unreliable.^14^ This limited outreach is due to the fact that AYA in the formal education system only represent 18–30% of their population,^20^ which underscores the need for inclusive, community-based approaches that extend beyond schools to engage out-of-school AYA and marginalised groups.

The heightened HIV burden among AGYW in Chad is influenced by socio-cultural norms, early marriages and gender-based violence,^10^,^12^ all of which restrict their access to education and healthcare. These structural barriers perpetuate a cycle of vulnerability and limited agency, emphasising the importance of tailored interventions that address both gender and age-specific needs.^21^ Additionally, discrimination and a lack of confidentiality in healthcare settings create a climate of fear that discourages AGYW from seeking care and adhering to treatment regimens.^13 14^

This study builds on prior research that highlighted sensemaking as a crucial process in how AYA interact with healthcare systems.^14^ Sensemaking, or the way individuals interpret and respond to complex situations, provides a framework for understanding the cognitive and social processes that guide AYA as they navigate SRH and HIV services.^22^ It involves constructing, deconstructing and reconstructing understandings to navigate daily information and stimuli.^23^ This framework is relevant in examining how people create narratives or mental maps to guide decision-making and actions in new or challenging circumstances.^24^ Unlike behaviour-focused theories, such as social cognitive theory or the theory of planned behaviour, sensemaking delves into interpretive processes that influence health decisions.^25 26^ Sensemaking focuses on individual and collective narratives, essential for understanding how young people perceive SRH and HIV issues amid societal stigma and cultural taboos.^22^ This approach is particularly relevant for Chad, where understanding the interplay between personal experiences and social contexts is critical. Its emphasis on collective narratives within peer groups and communities is crucial in SRH and HIV contexts where peer influence is significant.^27^ Beyond its application in organisational theory, sensemaking has been increasingly recognised as a valuable framework in healthcare contexts. For instance, Mamykina *et al* examined how individuals with chronic diseases make sense of their conditions and manage their health over time.^28^ Similarly, Künzler-Heule *et al* explored how men who have sex with men who are co-infected with HIV and hepatitis C virus make sense of their health status and navigate sexual risk reduction.^29^ Finally, Orgill *et al* focused on understanding how local health managers and staff make sense and contribute to innovative practices and solutions that address the challenges they face in their daily work.^30^

Previous research has shown that AYA in Chad often depend on social networks for SRH and HIV information, underpinned by trust and relationships.^14^ However, concerns about service quality, stigma and negative interactions with healthcare providers deter AYA from seeking necessary care.^13 14 21^ This study aims to explore how AYA access, interpret and make decisions regarding SRH and HIV care and how these processes influence their health-seeking behaviour. By examining these dynamics, this research contributes to the theoretical understanding of sensemaking in healthcare and offers practical insights for developing AYA-friendly services in Chad and similar settings.

## Methods

### Study design

**Figure 1:**
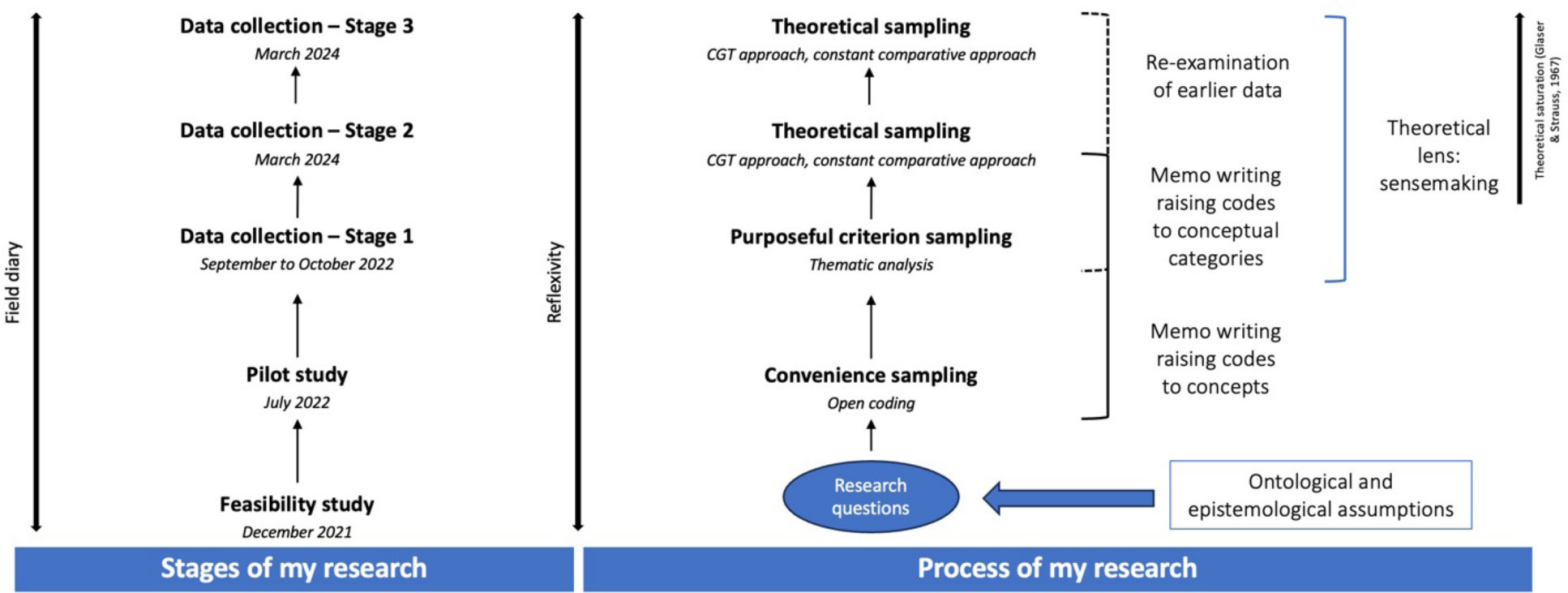
Stages and processes of the constructivist grounded theory approach employed for this study.

We applied a constructivist grounded theory (CGT) methodology, using sensemaking as a theoretical lens.^22 31^ Grounded theory is a qualitative research approach that seeks to develop theories directly from data rather than pre-existing hypotheses.^32 33^ It is particularly useful for exploring complex social processes that are not well understood.^32 33^ CGT was chosen because it allows for an in-depth exploration of how AYA access, interpret and navigate SRH and HIV services in Chad. Unlike traditional grounded theory, which assumes an objective reality that researchers uncover, or Straussian grounded theory, which follows a more structured set of coding procedures, CGT emphasises the co-construction of knowledge between researchers and participants within their social and cultural contexts.^31^ This means that findings are shaped by both the lived experiences of AYA and the researchers’ interpretations, making it a particularly suitable approach for studying youth perspectives in a dynamic and socially complex environment like Chad. This approach was essential in Chad’s multifaceted setting, as it enabled a nuanced understanding of AYA’s experiences through iterative data collection, reflexive engagement and constant comparison of emerging themes.^31 32^ Figure 1 illustrates the key stages of the grounded theory approach used in this study, adapted from Charmaz.^31 32^

### Sampling and recruitment

**Figure 2:**
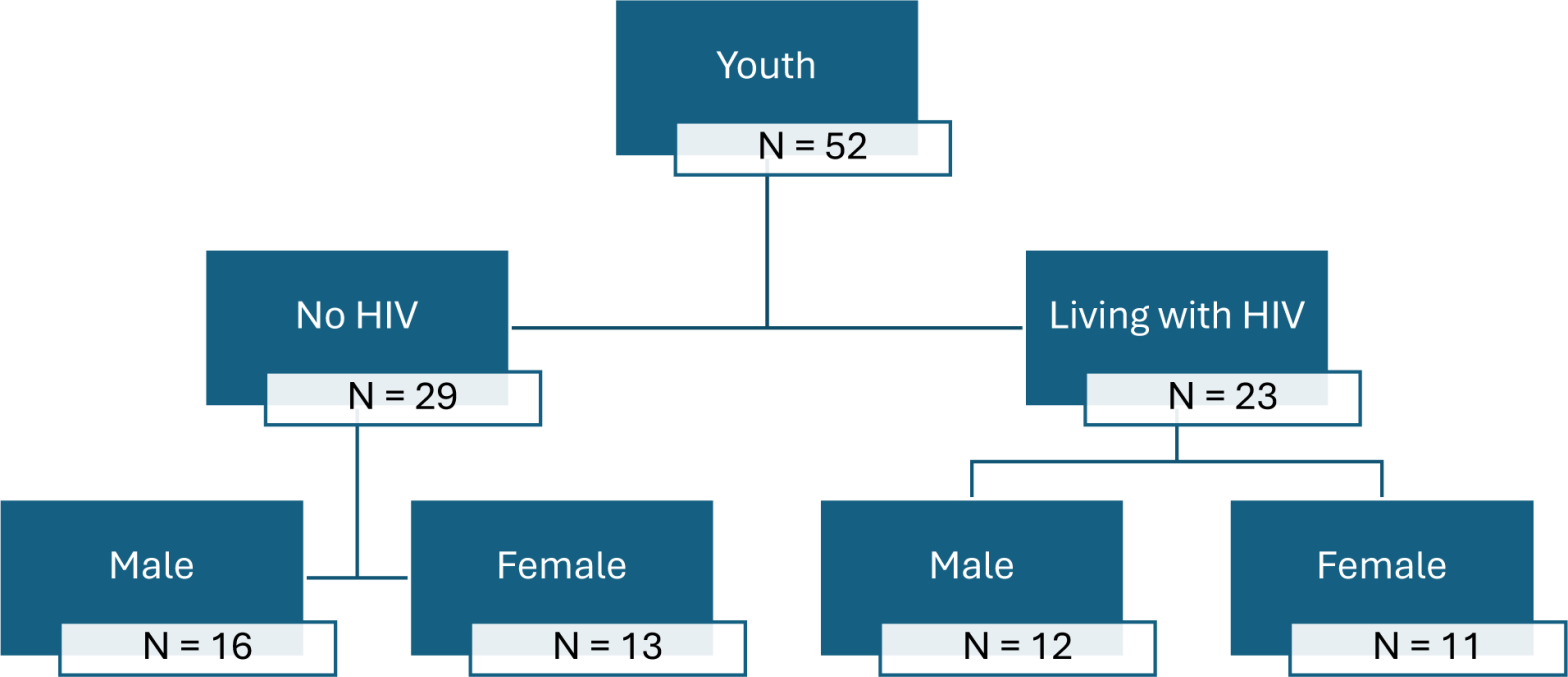
Flowchart of participants in the AYA focus group discussions.

As this study was part of a larger project involving AYA and healthcare providers, data collection occurred in three stages (figure 1) to comprehensively capture AYA (stages 1 and 2) and healthcare providers’ (stage 3) perspectives. However, this present study did not include stage 3. Prior to stage 1, we conducted feasibility and pilot studies to obtain insights into the study setting and type of participants needed for stage 1. Below is a description of each stage:

- Stage 1: 48 AYA were selected through convenience sampling to conduct four focus group discussions (FGDs; each consisting of 12 participants) to assess the contextual factors and mechanisms that influence access to information and health services,^14^ as well as understand AYA’s experience of HIV supportive norms in Chad.^21^
- Stage 2: building on findings from stage 1, sensemaking emerged as a helpful framework to answer our research question. Theoretical sampling was applied to focus the study on one province to capture both homogeneity and heterogeneity. Homogeneity was achieved by narrowing the age range and categorising participants by HIV status, while heterogeneity was ensured by increasing the number of FGD participants and stratifying them by age groups. Recruitment of AYA living with HIV included snowball sampling facilitated by the National Network of Associations of People Living with HIV (RNTAP+). Participants engaged in participatory activities, including a pile-sorting exercise, as part of the FGDs to elaborate on their sensemaking processes. The pile-sorting activity (online supplemental S1 file) involved participants writing reasons for seeking or avoiding SRH and HIV services on index cards, sorting them by influence and discussing their rationale. Typical FGDs include 6–10 participants, but since our study had participatory activities, we designed it to have 16 AYA in each group, divided into four subgroups of four. Eligible participants were AYA aged 15–24, and those willing to join were randomly selected from a provided list. The final sample included 52 AYA (aged 15–24) from N’Djamena, stratified by HIV status and gender to capture diverse experiences. Figure 2 presents the flowchart of participants.

### Data collection

As above-mentioned, analysing data from stage 1 (online supplemental S1 table) revealed that sensemaking was an effective framework, which we then used to inform the design of stages 2 and 3. We identified four themes for sensemaking: (1) making sense of SRH and HIV care, (2) expectations and knowledge, (3) previous care experiences and (4) prioritising choices. Using these themes, we developed a topic guide (online supplemental S2 file) for AYA-led participatory action research activities.^33^,^35^ AYA then actively engaged in FGDs and participatory exercises such as decision-making, community mapping and pile sorting to explore these themes in stages 2 and 3.^36^,^38^

A team of 14 locally trained data collectors conducted the focus groups and activities, ensuring gender alignment between study members and participants. FGD participants’ demographics are shown in table 1. Participants in each FGD were asked to come during their scheduled appointment, and their transportation expenses were reimbursed (not exceeding US$10). Sessions were held in French and Arabic in a secure private location of the Blue Cross Chad headquarters. All FGDs were audio-recorded with AYA’s consent and were transcribed verbatim. As FGDs were divided by themes, each theme took 90–120 min for a total of five themes (online supplemental S2 file). Each transcript was discussed with the entire team to ensure that all cultural nuances were captured.

**Table 1:** Sample demographics for adolescents and young adults

	N	%
Total	52	100
Sex		
Male	28	53.8
Female	24	46.2
HIV status		
HIV positive	23	44.2
HIV negative	29	55.8
Religion		
Muslim	5	9.6
Christian	47	93.4
Highest educational level		
No diploma	7	13.5
Enrolled in high school	28	53.8
High school diploma	11	21.2
Bachelor’s degree	6	11.5
Marital status		
Single	48	92.3
Married	4	7.7
	**Mean (SD)**	**Range**
Age	19.5 (0.90)	15–24

### Data analysis

#### Analysing sorting pile data

We began constructing a similarity matrix for each FGD based on the average scores of subgroups. For example, the male HIV group, divided into four subgroups, had four separate similarity matrices. The final matrix was calculated by averaging these four matrices to identify key themes related to why AYA seek or avoid SRH and HIV services (onlinesupplemental S3  S4 files).

#### Analysing focus group discussions

We used ATLAS.ti (V.22) to analyse the 13 FGDs following the CGT approach, following a CGT approach to systematically developing themes and a core category from participant narratives.^31 32 39^ Grounded theory provided an iterative process that allowed us to refine our understanding of how AYA navigate SRH and HIV services. The analysis followed three stages: initial, focused and theoretical coding.^31 32^ In the initial coding phase, three team members conducted line-by-line coding in French and Arabic, ensuring linguistic and cultural accuracy before translating results into English (online supplemental S5 file). This stage allowed for an open and detailed examination of participant responses, capturing key concepts without imposing predefined categories. Using a constant comparative approach, each new transcript was systematically compared against previously coded transcripts, allowing for ongoing refinement of emerging concepts. The focused coding phase involved organising the initial codes into broader categories, emphasising commonalities and variations across participants. Two members refined and structured these subcategories through continuous discussion and reanalysis (online supplemental table 2). Codes that were conceptually similar were grouped together, ensuring that the analytical framework remained rooted in participant experiences. Data saturation was reached by transcript n=10, as no substantially new information emerged, but analysis continued across all transcripts to capture age-related variations (ages 15–24 years) within the sample. Finally, theoretical coding synthesised these subcategories into broader themes, establishing a coherent theoretical model that captured the core dimensions of AYA’s healthcare decision-making processes. From these patterns, we identified a core category, ‘empowering AYA in health decision-making through adaptive sensemaking’. This core category encapsulates how AYA interpret, navigate and act on health information based on their personal experiences, social interactions and systemic constraints. The research team reviewed the final coding structure and thematic model through iterative discussions, ensuring consensus on the interpretation of findings.

### Research trustworthiness

To ensure trustworthiness, we adhered to Lincoln and Guba’s criteria: credibility, transferability, dependability and confirmability.^40^ Credibility was strengthened through the research team’s active engagement in data collection and analysis, supported by locally trained interviewers familiar with local customs. Transferability was achieved by thoroughly documenting our methods and contextualising participants’ stories while protecting their anonymity. Dependability was maintained by establishing coding rules through team consensus. Confirmability was reinforced through self-reflection, memo writing and peer debriefings to identify and mitigate potential biases.

### Positionality statement

Our research team was both diverse and multinational, proficient in various local languages and disciplines. As a member of this team and a native of Chad, I brought my understanding of local challenges and cultural sensitivity into the research process. Being closely aligned with the AYA demographic, I was able to approach the study with a nuanced perspective of the issues affecting young people in Chad. The research team included researchers and social scientists from Harvard University, the Centre for Research in Anthropology and Human Sciences, Blue Cross Chad, RNTAP^+^ and the Ministry of Public Health and Prevention. This diverse composition helped to address potential biases through collaborative engagement and cultural insight.

The inclusion of locally trained interviewers further enriched our analysis by capturing cultural nuances and facilitated acceptance within the community. This aspect of the research was reflexive and collaborative, engaging continuously with RNTAP^+^ and Blue Cross Chad partners. Despite the team’s diverse composition, potential biases in the conclusion could not be entirely ruled out. To address this, triangulation was applied, involving sharing findings with AYA and young people not interviewed and experts on the subject to identify possible inaccuracies or biases overlooked by the research team.

### Ethical considerations

Informed consent forms were read aloud by the interviewers before the FGDs began. Participants had the opportunity to ask questions during the consent process and gave written consent or assent for participants under 18 years old.

For minors, parents were contacted and the purpose of the study was explained. To reduce the overall burden on parents, the IRB found it would be most appropriate to waive documentation of parental permission and obtain parental permission exclusively verbally via phone call. The research did not involve participants in its design, conduct or reporting due to practical and ethical limitations.

## Results

**Figure 3:**
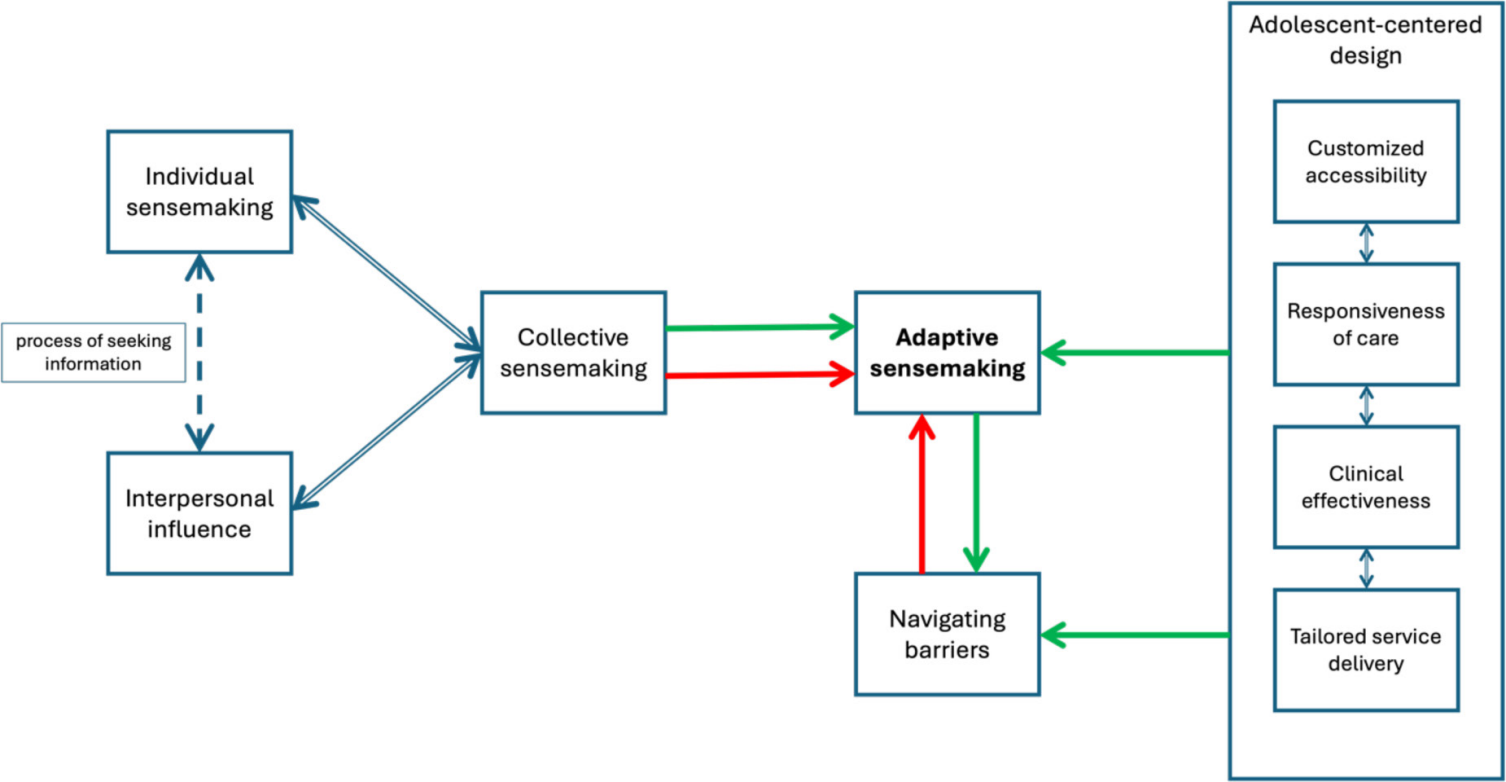
Theoretical model of empowerment of the youth in health decision-making through adaptive sensemaking.

Our core category, ‘empowering AYA in health decision-making through adaptive sensemaking’, is built on three themes: (1) collective sensemaking, (2) navigating barriers and (3) systemic accessibility through an AYA-centred design. The derivation of a core theme or category emerges through constant comparison—a hallmark of CGT—where data, codes and categories are continually compared against new data to refine their meaning and relevance.^37 38^ The core theme is the category that demonstrates significant explanatory power and appears consistently throughout the data. It integrates-related subcategories, linking them to illustrate a comprehensive understanding of the studied phenomenon. Therefore, the core category identified, ‘empowering AYA in health decision-making through adaptive sensemaking’, serves as an overarching theme that encompasses these subcategories. This core category—‘this theory’—emphasises the need for an adaptive sensemaking approach that integrates personal experiences, social influences and systemic factors to support AYA in managing their health decisions (figure 3). Considering these aspects in policy and interventions can enhance healthcare engagement, increase service use, improve treatment adherence and inform service redesign to be more effective and AYA-centred. Detailed findings and coding structures are provided in the online supplemental S3 table and onlinesupplemental S3  S4 files. Three themes and six constituent subthemes emerged from the grounded theory analysis as shown in table 2.

**Table 2:** Themes and subthemes for AYA focus group discussions

Themes	Subthemes	Participants* (n=52)	FGDs† (n=13)	Codes (n)
Collective sensemaking	Individual sensemaking	45	13	22
	Process of seeking information	13	6	6
	Interpersonal influence	24	7	8
Navigating barriers	Trust and safety	40	13	17
	Challenges in HIV care	25	7	10
Systemic accessibility and quality through an AYA-centred design	Customised accessibility	44	12	5
	Responsiveness of care	44	11	8
	Clinical effectiveness	44	12	4
	Tailored service delivery	44	12	8

* Total number of participants who mentioned the [(subtheme]).

† Total number of FGDs in which the [(subtheme]) was mentioned.

AYAadolescents and young adultsFGDfocus group discussion

The roadmap for this section is as follows: we begin with an explanation of collective sensemaking, showcasing how AYA construct and share health knowledge within their social networks. This is followed by an exploration of the barriers AYA face when navigating healthcare, including psychological, logistical and financial challenges. Lastly, we discuss systemic accessibility and the significance of designing AYA-friendly, AYA-centred services. Throughout, we clarify the distinctions between categories and themes to maintain consistency and provide a coherent narrative linking the methodology to the findings.

### Theme 1: collective sensemaking

Collective sensemaking emerged as a critical tool for AYA to navigate the complexities of SRH and HIV by transforming individual insights into shared understanding through peer and family interactions. AYA described ‘sharing everything in words*’* (Participant #4, age range 20–22) and meeting regularly at social clubs to discuss ‘medication and advice’ (Participant #39, age range 20–22). These conversations, guided by peers and healthcare providers, integrated diverse perspectives, making information more relatable and actionable. As one participant noted, ‘In clubs, we advise each other and have open discussions’ (Participant #1, age range 20–22). This dialogue enabled a communal adaptability, where individual experiences were synthesised into a broader, evolving group narrative.

#### Subtheme 1: individual sensemaking

Individual sensemaking began with personal experiences like ‘feeling unwell’ (Participant #51, age range 20–22) or realising the risks of ‘unprotected sex’ (Participant #34, age range 18–19), prompting AYA to seek health information or care. One AYA shared, ‘After sex without a condom, my girlfriend hadn’t had her period, so we went for a test’ (Participant #1, age range 20–22). For AYA living with HIV, the focus was on managing their health and finding ‘moral support’ (Participant #32, age range 20–22). Conversely, HIV-negative AYA emphasised prevention, as one noted, ‘When I found out it was negative, it prompted me to take preventive measures’ (Participant #4, age range 20–22).

The initial recognition of health concerns set the path for future decisions, shaped by internal reflections and weighing the severity of symptoms against their beliefs and access to care. As one AYA shared, ‘I think it’s better to go every month, but for lack of means, it’s not possible. Illnesses can become chronic’ (Participant #3, age range 23–24), highlighting the ongoing struggle with consistent care access. These narratives highlighted the nuanced and subjective nature of individual sensemaking in the realm of AYA SRH and HIV care, illustrating how personal experiences and interactions with healthcare influence their understanding and actions regarding their health.

#### Subtheme 2: interpersonal influence

Interpersonal influence was crucial in AYA health decision-making, as they sought advice and validation from trusted sources. Elders and family members, especially those in healthcare, were frequently consulted for credible guidance. One AYA noted, ‘I take advice from my elders before going to the hospital’ (Participant #4, age range 20–22), reflecting the trust placed in family wisdom. Similarly, parental support also provided emotional backing and practical help, as illustrated by, ‘Taken by my dad for the test…he came back to get the results’ (Participant #26, age range 18–19). This subtheme focuses on the role of interpersonal connections as sources of emotional support and practical advice, emphasising how such relationships guide AYA choices. The reliance on family members with medical knowledge was particularly noteworthy.

It’s when I feel sick, but my aunt is a nurse, so I ask her to take me to the hospital…when I feel bad, she’s the one who take care of me. (Participant #1, age range 20–22)

Pharmacists were also valued for their accessibility and specific advice. As one AYA shared, ‘At least the pharmacists can direct us to go to the doctors’ (Participant #4, age range 20–22), demonstrating how guidance from different trusted figures influenced decision-making.

#### Subtheme 3: process of seeking information

The process of seeking information was an intentional and critical step undertaken by AYA, aimed at bridging the knowledge gaps that emerged during the initial sensemaking phase. This subtheme is distinguished by the active efforts AYA make to gather information from a range of sources, including healthcare professionals, family members, peers and community networks:

My grandparents took me to the hospital because I was ill. They said it was the illness that killed my parents. (Participant #39, age range 20–22)

My sources of information included the Association of People Living with HIV, radio, newspapers, and television. (Participant #23, age range 20–22)

The emphasis here is on the proactive pursuit of information rather than seeking validation or emotional support. The quality and accessibility of information greatly influenced healthcare decisions: ‘I resign myself to the pharmacy level because at least the pharmacist can direct us to go to the doctors’ (Participant #4, age range 20–22). Financial barriers were also a major concern, as expressed by a participant: ‘Hospitals are expensive for examinations, and due to a lack of resources, we can’t go there’ (Participant #1, age range 20–22). This iterative information-seeking process allowed AYA to refine their decisions and adapt their healthcare-seeking strategies based on new insights and changing needs.

### Theme 2: navigating barriers

AYA faced multiple barriers when seeking SRH or HIV care, including psychological, financial and logistical challenges. Psychological barriers, like fear of an HIV diagnosis, prevented AYA from getting tested. One shared, ‘I was afraid of the truth… It can be traumatic, so it’s better to avoid the truth and live in the dark’ (Participant #4, age range 20–22). Financial constraints were also significant, as noted by a participant: ‘The difficulty is that I have no money to pay for medication’ (Participant #44, age range 20–22). Logistical issues, such as service availability and scheduling conflicts, added further obstacles, making it difficult for AYA to access the care they needed.

#### Subtheme 1: trust and safety

Trust and safety were critical factors in the healthcare decision-making process for AYA. Trust encompassed the credibility and reliability of the healthcare provider and the integrity of the community actor, as shown here: ‘My sources of advice are doctors, peer educators, and psycho-social counselors’ (Participant #42, age range 15–17). Environments that respected privacy and confidentiality were particularly valued, as reflected by a participant who noted, ‘At the end of each month, we meet at the social clubs for discussions and advice on the medication we take*.* The club’s name is “Only Family”’ (Participant #38, age range 18–19).

Safety extended beyond physical spaces to emotional support, where AYA felt free from judgement. One participant highlighted that specialised facilities provided ‘good care’ and were ‘treated well, with no stigma’ (Participant #50, age range 15–17), showing how stigma-free environments are vital for building trust. Another AYA emphasised the need for ‘being courteous and taking good care of patients’ (Participant #43, age range 23–24), illustrating the impact of provider behaviour on trust and comfort.

#### Subtheme 2: challenges in HIV care

AYA faced multifaceted challenges in HIV care, involving not just the individual’s perception and experiences but the broader healthcare system’s responsiveness to their needs. Financial constraints were a significant hurdle, which not only limited access to necessary treatments, but also contributed to delays in seeking care, which could have severe implications for health outcomes.

Hospitals are expensive for examinations and due to a lack of resources, we can’t go there. If rates can be reduced for us young people, it would be good because we do not have the financial means. (Participant #1, age range 20–22)

Moreover, psychological barriers, such as the fear of stigma or the diagnosis itself, played a pivotal role in deterring AYA from accessing HIV care. This fear was compounded by concerns about privacy and confidentiality within healthcare settings, which were crucial for AYA dealing with HIV:

I was afraid of the truth…It can be traumatic, so it’s better to avoid the truth and live in the dark. (Participant #4, age range 20–22)

Geographical proximity and the accessibility of healthcare settings also emerged as challenges, with AYA emphasising the importance of ‘proximity’ (Participant #5, age range 18–19) and ‘accessibility*’* (Participant #10, age range 22–24) in their care pathways. The ease of accessing healthcare services, influenced by physical distance and the availability of AYA-friendly facilities, was critical in determining whether AYA would seek out and engage with HIV care.

The attitude and professionalism of healthcare staff were often found to be inadequate, presenting a significant challenge for AYA. One participant noted, ‘In public hospitals, the staff does not treat us well sometimes. They neglect and stigmatise us’ (Participant #44, age range 20–22 years), emphasising the negative impact of poor treatment on their willingness to seek care. Another shared, ‘The staff could improve their service by treating and giving more listening time to patients’, (Participant #26, age range 18–19) highlighting the need for more empathetic and respectful interactions. Despite these challenges, AYA valued ‘professionalism’ (Participant #47, age 18–19) and a positive ‘attitude’ (Participant #36, age 18–19), underscoring that the absence of these qualities hindered their engagement with HIV services.

### Theme 3: an AYA-centred design

An AYA-centred design emerged as essential for enhancing the quality of SRH and HIV care and facilitating the uptake of services among AYA. This approach, envisioned through participant feedback and analysis, underscored the importance of creating healthcare environments that are accessible, welcoming and sensitive to AYA needs. It emphasised the value of professionalism, positive attitudes and the educational role of staff to make AYA feel respected and cared for. The goal of integrating evidence-based care with a patient-centred approach was to address both medical and psychosocial aspects of AYA health. By focusing on these elements, such a design would help reduce financial, psychological and logistical barriers, fostering a healthcare experience that is effective, supportive and empowering for AYA.

#### Subtheme 1: customised accessibility and tailored service delivery

Customised accessibility in AYA-centred care focused on tailoring healthcare environments to meet AYA-specific needs and preferences. This included creating welcoming spaces and streamlined services that resonated with young individuals. One participant shared, ‘I go to the health centres to be consulted by nurses, then I’m referred to hospitals for tests’ (Participant #3, age 23–24), emphasising the need for settings that simplify navigation and are tailored to AYA, making it easier for them to engage with health services.

#### Subtheme 2: responsiveness of care

Responsiveness in AYA-centred care focused on providers’ ability to adapt, engage and empathise with AYA, ensuring services meet their unique needs. This required not only following clinical guidelines but also understanding the psychosocial complexities of AYA health. One AYA noted, ‘My doctor is very available, and I can contact at any time’ (Participant #4, age 20–22), highlighting the importance of continuous support and recognising their vulnerability. Another emphasised the significance of empathy, saying, ‘I want my doctor to be neutral but empathetic. Courtesy is an important element for the doctor to build the patient’s confidence’ (Participant #26, age 18–19). Family involvement is also vital, as shown by one participant’s reflection: ‘My mother did it [HIV test] when I was 5, and still goes with me for my viral load tests’ (Participant #33, age 18–19), underscoring the supportive role that trusted family members can play. AYA-centred services should facilitate the involvement of these trusted support systems or create linkages to external support networks when such personal support is unavailable. Additionally, linkage to support networks, such as ‘Psycho-social counsellors advise us, and I rely on the associations of people living with HIV for support’ (Participant #33, age 18–19), is crucial for comprehensive care. Such responsive care, built on trust, empathy and ongoing engagement, must integrate clinical expertise and personal connection to empower AYA in their health decisions.

#### Subtheme 3: clinical effectiveness

Clinical effectiveness in AYA-centred care ensures that interventions are both evidence-based and tailored to AYA-specific needs. This requires employing best practices proven to work for AYA. One participant suggested, ‘Each district should have a health center specifically for AYA offering all services for SRH and HIV care’ (Participant #31, age 15–17), highlighting the need for specialised services. Additionally, AYA valued varied sources of advice, such as ‘health personnel in educational establishments’ (Participant #25, age 15–17) and ‘private health centers on social networks’ (Participant #23, age 15–17), underscoring the need for a comprehensive, multifaceted approach that supports their overall well-being.

## Discussion

The findings emphasise that adaptive sensemaking serves as both cognitive and social process through which AYA interpret, navigate and respond to SRH and HIV challenges, ultimately shaping their health decisions. This process is influenced by the availability and accessibility of youth-friendly healthcare systems, as well as peer and community support structures, which together create an enabling environment that empowers AYA to make informed choices and engage with health services more effectively. The study highlights how collective sensemaking, navigating barriers and systemic accessibility collectively contribute to how AYA process information and make decisions related to their health. Understanding and integrating these factors into service design can enhance engagement, service utilisation and treatment adherence among AYA.

The findings of this study provide meaningful contributions to the existing body of knowledge on AYA healthcare, particularly in SRH and HIV care. AYA in Chad face significant barriers to SRH and HIV care, including psychological fears (eg, fear of an HIV diagnosis), financial constraints and logistical challenges. These barriers are consistent with findings from studies highlighting similar issues across other SSA countries.^41^,^45^ For instance, Nyblade *et al* underscored how stigma within health clinics can impede pre-exposure prophylaxis (PrEP) access and use among AGYW in South Africa, emphasising conflicting perspectives on care.^41^ Similarly, studies such as those by Enane *et al* (2023) in western Kenya and Langat *et al* in the coastal counties of Kenya reported that stigma, logistical challenges and economic constraints severely impacted AYA engagement in HIV care.^42 43^ Confidentiality concerns and fear of judgement were identified as major deterrents to service uptake, underscoring the necessity for healthcare workers to be continuously trained in effective communication to create a supportive and non-judgemental environment. This necessity is echoed in the work of Mengesha *et al* (2023), which emphasised that disclosure of HIV status is critical for antiretroviral therapy (ART) adherence but hindered by fears of stigma and judgement.^44^ Nyirenda *et al* in Zambia further highlighted that stigma, fear and inadequate provider attitudes contribute significantly to gaps in care for AYA.^45^ This aligns with the findings of a study that examined the perspectives of healthcare providers on the provision of PrEP to AGYW in Kenya, South Africa and Zimbabwe, which found that healthcare providers needed to be trained in effective communication techniques to better discuss PrEP and sexual health.^46^ Addressing these barriers requires a redesign of services centred on AYA needs, as emphasised by other research,^47 48^ including the study in Gauteng, South Africa, which highlighted the importance of youth-friendly, approachable staff and the integration of private, confidential service spaces.^49^ Such studies advocate for training healthcare workers in effective communication and integrating community actors like peer educators to create an environment that encourages AYA engagement in SRH and HIV care.

Our findings emphasise the importance of trust, safety and tailored services in creating AYA-friendly healthcare spaces. This is consistent with previous research which shows that AYA are more likely to engage with healthcare services when they feel respected, and their privacy is protected.^4350^,^53^ AYA prefer engaging with community actors over healthcare workers for support, underscoring the need to integrate these roles within healthcare settings. WHO recommends involving community actors such as peer counsellors, expert patients and mentor mentors for AYA living with HIV,^51^ as successful models have shown improved engagement and adherence.^5254^,^56^ Although these community actors exist in Chad, they have not been widely deployed.^12^ In our study, healthcare workers and community actors employed various strategies focused on education, emotional support, building trust and creating a conducive environment to improve adherence to treatment among AYA. With AYA (aged 15–24) ART coverage at 47%, integrating these community actors into AYA-friendly spaces can facilitate the adaptive sensemaking process among AYA, thereby improving service delivery in Chad.^3 57^

The findings reveal notable distinctions between adolescents (aged 15–19) and youth (19–24). Adolescents often needed family support and faced higher vulnerability to stigma. In contrast, youth demonstrated greater autonomy but encountered significant financial hurdles. These age-related differences suggest that interventions must be tailored to address the unique needs of each group. Furthermore, the limited insights into university-level programming indicate a gap in services tailored for higher education students, highlighting the need for targeted SRH and HIV interventions within university settings. Minimal involvement from the private sector, with participants favouring public and community services despite noted challenges, underscores the importance of strengthening both public and private sector partnerships to create comprehensive and accessible AYA-friendly services.

Comparing these results to previous studies, it is evident that AYA-centred care must combine medical and psychosocial support. Past HIV interventions in Chad focused mainly on biomedical services. This study shows that emotional and social support are equally crucial for AYA, suggesting that adaptive sensemaking—where experiences, influences and systemic factors intersect—empowers informed decisions and better health outcomes.

### Implications for research, policy and practice

This study underscores the need for an AYA-centred approach in research, policy and practice that goes beyond biomedical interventions to address the psychosocial and systemic factors shaping AYA health behaviours. Within Chad’s healthcare system, which often faces constraints such as limited infrastructure and accessibility issues, these findings emphasise the importance of tailoring services to different levels of care, including primary and community health facilities where AYA seek support. Health system decision-makers should focus on strategies that extend beyond training healthcare workers to include strengthening the infrastructure of healthcare settings, streamlining services and ensuring consistent quality of care across various levels of the health system.

For research, integrating mixed-method approaches, such as discrete choice experiments, can quantify AYA preferences and provide deeper insights into the attributes that drive engagement with SRH and HIV services. While this study was conducted with a specific cohort, the implications are broadly relevant to similar low-resource settings where AYA face multifaceted barriers to care. These approaches could inform research in other SSA contexts with analogous healthcare challenges.

Policy implications include the development of AYA-inclusive strategies that incorporate the voices of AYA and community actors in the design and delivery of health services. Policymakers should prioritise the creation of AYA-friendly, accessible and confidential healthcare environments that consider logistical and financial barriers and align with Chad’s current healthcare framework. Health system decision-makers should explore partnerships with community and private sector stakeholders to bolster service capacity and broaden outreach efforts.

For practice, frontline healthcare workers should be trained in effective communication and cultural competency to build trust and offer personalised support. Additionally, integrating community actors such as peer educators and counsellors into health service delivery can bridge the gap between formal healthcare settings and the lived experiences of AYA, thereby enhancing engagement and adherence to treatment. This approach is not limited to Chad; it can be adapted for similar settings where health systems face challenges in reaching and engaging AYA. Together, these measures can foster a more responsive and supportive healthcare ecosystem for AYA, ensuring that the broader health system becomes more inclusive and effective in addressing the unique needs of this population.

### Strengths and limitations

A key strength of this study is its use of an AYA-centred approach, incorporating AYA voices through participatory methods to capture nuanced perspectives on SRH and HIV care. The use of locally trained interviewers ensured cultural sensitivity and enhanced the credibility of the findings, while the CGT methodology allowed for a rich exploration of AYA experiences. Additionally, the integration of community actors in the research design provided insights into the broader sociocultural dynamics influencing AYA health behaviours.

However, several limitations should be noted. First, the study focused primarily on the general AYA population, potentially overlooking the specific needs of marginalised groups such as lesbian, gay, bisexual, transgender and queer (LGBTQ+) AYA, sex workers and young people who inject drugs. Actively recruiting these key populations through community partnerships and specialised focus groups would provide a more comprehensive view. Second, the qualitative nature of the study, while offering in-depth insights, limits the generalisability of findings. Self-reported data may also introduce bias, as participants might under-report sensitive information or over-report desirable behaviours. Third, the study was geographically limited to specific regions, which may not represent the full spectrum of AYA experiences across Chad. Expanding the study to include diverse and underserved areas would enhance the depth of understanding. Fourth, the exclusion of parents from the research limits insights into family dynamics that impact AYA health-seeking behaviour. Additionally, the study did not thoroughly examine the influence of digital and online health information, which is increasingly relevant for young people. Lastly, involving AYA as co-researchers or advisory panel members could ensure the findings reflect their perspectives and are more actionable, enhancing the overall relevance and inclusivity of the research.

## Conclusion

This study highlights adaptive sensemaking as both a cognitive and social process that enables AYA in Chad to interpret health-related information, assess risks and make informed SRH and HIV decisions. However, this process is shaped by individual experiences, interpersonal influences and systemic barriers, requiring a more responsive and supportive healthcare environment. Empowerment occurs when health systems acknowledge and integrate AYA’s sensemaking processes into service design, ensuring care is accessible, inclusive and stigma-free. Our findings suggest that an adaptive sensemaking framework is essential for engaging AYA in HIV care, particularly within Chad’s resource-constrained healthcare system, where many AYA rely on public and community-level facilities with limited capacity. Implementing youth-centred service redesigns, integrating peer and community support structures and improving provider training can enhance engagement, service utilisation and adherence to treatment.

Aligning AYA health services with universal health coverage goals requires financial protections, decentralised service delivery and participatory approaches that empower AYA to navigate and engage with healthcare effectively. Beyond HIV and SRH, these insights apply to mental health and non-communicable diseases, where stigma, accessibility and lack of youth-friendly services remain persistent challenges. By embedding participatory action research principles, this study not only advances theoretical discourse on sensemaking in healthcare but also offers practical insights for health interventions and policies tailored to Chad’s context. Developing AYA-centric health services that leverage sensemaking processes is essential for designing inclusive, accessible and supportive care environments that address logistical and financial barriers. Strengthening these approaches will empower AYA and ultimately lead to better health outcomes across multiple dimensions of AYA well-being.

## supplementary material

10.1136/bmjgh-2024-017763 online supplemental file 1

10.1136/bmjgh-2024-017763 online supplemental file 2

10.1136/bmjgh-2024-017763 online supplemental file 3

10.1136/bmjgh-2024-017763 online supplemental file 4

10.1136/bmjgh-2024-017763 online supplemental file 5

10.1136/bmjgh-2024-017763 online supplemental table 1

10.1136/bmjgh-2024-017763 online supplemental table 2

10.1136/bmjgh-2024-017763 online supplemental table 3

## Data Availability

No data are available.
