# Supplementary material for: Optimising adolescents and young adults’ utilisation of sexual and reproductive health and HIV services in Chad: a sensemaking approach
Source: BMJ Glob Health. 2025 Mar 26;10(3):e017763. doi: 10.1136/bmjgh-2024-017763 (PMC11950941; doi:10.1136/bmjgh-2024-017763)
Supplement: online supplemental file 1 [file bmjgh-10-3-s001.pdf]

S1 File: Examples from a pile-sorting activity in a focus group discussion (FGD).

In the pile-sorting activity conducted within the FGDs (S2 File; question 7, theme 4 group activity), AYA were prompted to reflect on personal experiences or hypothetical scenarios related to seeking sexual and reproductive health (SRH) or HIV services. They began by thinking about a time when they needed such services and either decided to go or, if they had never sought them, imagined doing so. Each participant wrote down reasons for seeking the services on separate index cards, then prioritized these factors from most to least influential. Next, they reflected on a situation where they needed SRH or HIV services but chose not to go, writing down reasons for this decision on index cards and prioritizing them similarly. After prioritizing, participants articulated the meaning of their card piles and shared their reasoning with the group. This activity allowed for a deeper exploration of the motivations, barriers, and priorities influencing adolescents' decisions, providing valuable insights into how they make sense of and navigate SRH and HIV services. Below are examples of a pile-sorting activity within a FGD:

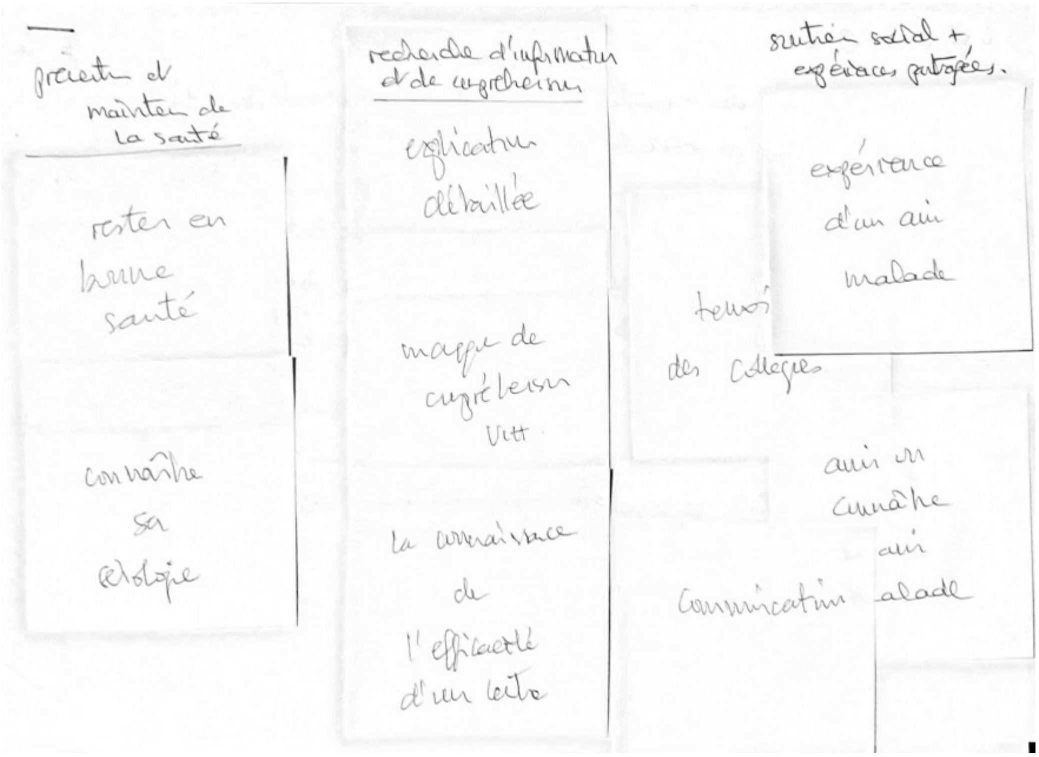

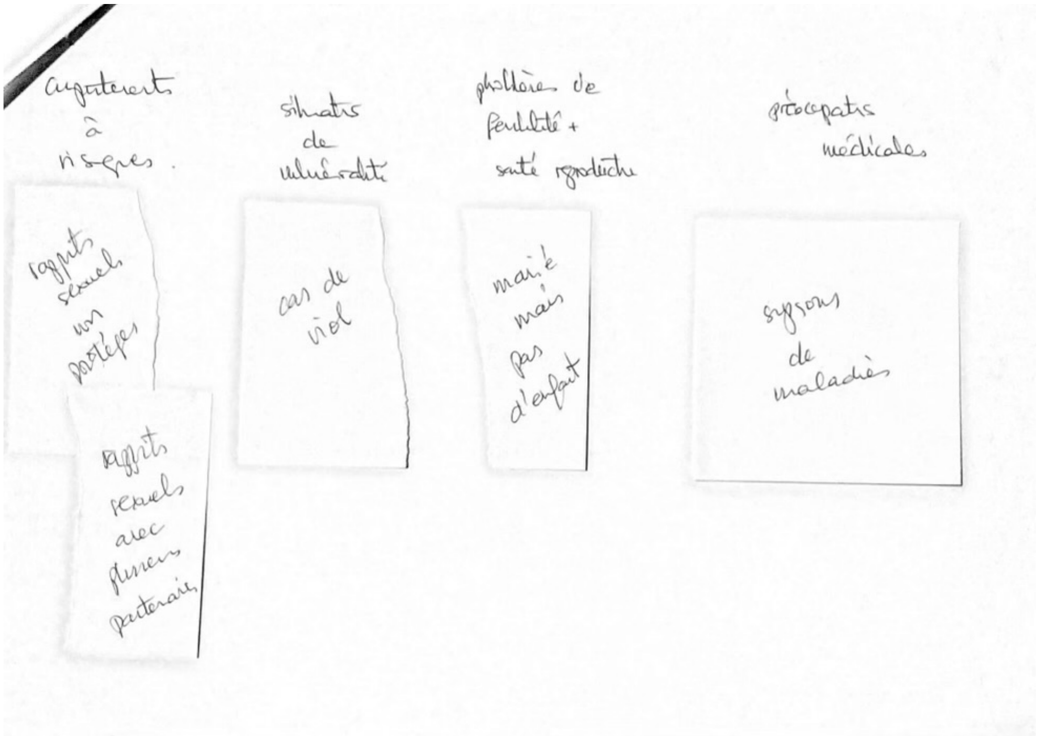
comportements à risques.
situations de vulnérabilité
problèmes de fertilité + santé reproductive
préoccupations médicales
rapports sexuels non protégés
rapports sexuels avec plusieurs partenaires
cas de viol
marié mais pas d'enfant
suspicions de maladies
