# Supplementary material for: Optimising adolescents and young adults’ utilisation of sexual and reproductive health and HIV services in Chad: a sensemaking approach
Source: BMJ Glob Health. 2025 Mar 26;10(3):e017763. doi: 10.1136/bmjgh-2024-017763 (PMC11950941; doi:10.1136/bmjgh-2024-017763)
Supplement: online supplemental file 3 [file bmjgh-10-3-s003.pdf]

S3 File: Summary of results for pile sorting activities regarding access to care.

For a similarity matrix, a value of 1 indicates maximum similarity, while values closer to 0 indicate lower similarity. The values between 0 and 1 represent varying degrees of similarity based on the subjective assessment of the conceptual closeness between the themes. A score above 0.7 or 0.8 might be considered to reflect high similarity, whereas a score below 0.3 or 0.2 are often considered low similarity.

When comparing based on HIV status and further stratifying by gender, we observed distinct themes and priorities that reflect their unique concerns, motivations, and needs regarding SRH and HIV services.

For HIV negative male and female participants, below is the similarity matrix:

Themes		1	2	3	4	5	6	7	8	9	10	11
1	Psychological support	1.0	0.0	0.0	0.0	0.0	0.0	**0.7**	**0.8**	0.0	0.0	0.0
2	Physical health issues	0.0	1.0	0.0	0.0	0.0	0.0	0.0	0.0	0.6	0.0	**0.8**
3	Cause of concern	0.0	0.0	1.0	0.0	0.0	0.0	0.0	0.0	**0.9**	0.0	0.0
4	Consequence awareness	0.0	0.0	0.0	1.0	0.0	0.0	0.0	0.0	0.0	0.6	0.0
5	Prevention	0.0	0.0	0.0	0.0	1.0	0.0	0.0	0.0	0.0	0.0	0.0
6	Information and understanding	**0.7**	0.0	0.0	0.0	**0.7**	1.0	**0.7**	0.5	0.0	0.0	0.0
7	Social support and experiences	**0.8**	0.0	0.0	0.0	0.5	0.0	1.0	0.0	0.0	0.0	0.0
8	Risk behaviors	0.0	0.6	**0.9**	0.0	0.0	0.0	0.0	1.0	0.0	0.0	0.0
9	Vulnerability situations	0.0	0.0	0.0	0.0	0.0	0.0	0.0	0.0	1.0	0.5	0.0
10	Fertility and reproductive health	0.0	0.0	0.0	0.6	0.0	0.0	0.0	0.0	0.5	1.0	0.0
11	Medical concerns	0.0	**0.8**	0.0	0.0	0.0	0.0	0.0	0.0	0.0	0.0	1.0

From this similarity matrix, the following themes were identified:

- **Common motivators or barriers** emerged from the high similarity score between "psychological support" and "social support and experiences", suggesting that seeking emotional or psychological support was a common motivator for individuals considering SRH or HIV services.
- **Shared concerns and needs** emerged from the high similarity score between "physical health issues" and "medical concerns", highlighting that concerns about physical health and specific medical issues were prevalent reasons for seeking services.
- **Risk perception and behavior** emerged from the high similarity score between "risk behaviors" and "cause of concern", indicating that engaging in risky behaviors was a significant reason for seeking SRH and HIV services, driven by a recognition of these behaviors as potential causes for health concerns.
- **Influence of social networks** emerged from the high similarity score between "social support and shared experiences" and "prevention and health promotion", revealing the impact of peer networks and social testimonies on individuals' decisions to seek services.
- **Situational vulnerabilities** emerged from the high similarity score between "situations of vulnerability" and "fertility and reproductive health problems", highlighting the role of specific life situations (e.g., experiences of violence or fertility issues) in driving service-seeking behavior.

For HIV positive male participants, below is the similarity matrix:

Themes		1	2	3	4	5	6	7	8
1	Persistent illness	1.0	0.2	**0.7**	0.4	0.5	0.1	0.3	0.2
2	Fear of pregnancy	0.2	1.0	0.3	0.5	0.6	0.2	**0.7**	0.4
3	Multiple sexual partners	**0.7**	0.3	1.0	0.4	0.5	0.3	0.6	0.3
4	Screening	0.4	0.5	0.4	1.0	**0.8**	0.5	0.4	0.3
5	Health aspiration	0.5	0.6	0.5	**0.8**	1.0	0.6	0.5	0.5
6	Stock up on condoms	0.1	0.2	0.3	0.5	0.6	1.0	0.2	0.1
7	Unsafe sex	0.3	**0.7**	0.6	0.4	0.5	0.2	1.0	**0.8**
8	Wanting to feel safe	0.2	0.4	0.3	0.3	0.5	0.1	**0.8**	1.0

From this similarity matrix, the following themes were identified:

- **Proactive health guardianship** emerged from the high similarity score between "screening" and "health aspiration", suggesting that individuals who were interested in screening services were likely motivated by a broader desire to maintain or ensure their health.
- **Seeking security in vulnerability** emerged from the high similarity score between "unsafe sex" and "wanting to feel safe", suggesting that engaging in unsafe sex may have led to feelings of vulnerability or anxiety regarding potential health consequences, driving a strong desire for safety and security.
- **Consequence awareness and prevention** emerged from the high similarity score between "unsafe sex" and "fear of pregnancy", underscoring the direct connection between the behavior and its potential consequence.

For HIV positive female participants, below is the similarity matrix:

Themes		1	2	3	4	5	6	7	8
1	Get advice	1.0	0.0	**0.8**	0.0	0.0	0.0	0.0	0.0
2	Take care of yourself	0.0	0.0	0.0	0.0	**0.7**	**0.7**	0.0	0.0
3	Good information	**0.8**	0.0	0.0	0.0	0.0	0.0	0.0	0.0
4	Receive proper treatment	0.0	0.0	0.0	0.0	**0.8**	**0.8**	0.0	0.0
5	To take my ARV medication	0.0	**0.7**	0.0	**0.8**	0.0	0.0	0.0	0.0
6	To collect my treatment	0.0	**0.7**	0.0	**0.8**	0.0	0.0	0.0	0.0
7	To be followed by my doctor	0.0	0.0	0.0	0.0	0.0	0.0	0.0	**0.8**
8	To check my viral load	0.0	0.0	0.0	0.0	0.0	0.0	**0.8**	0.0

From this similarity matrix, the following themes were identified:

- **Information seeking and guidance** emerged from the high similarity score between "get advice" and "good information", suggesting that seeking advice and obtaining good information were closely in the context of health services.
- **Proactive health management** emerged from the high similarity score between "take care of yourself", "to take my ARV medication", and "to collect my treatment', suggesting that for individuals managing HIV, the concept of proper treatment was tightly bound to the specific actions of medication adherence and access.
- **Treatment adherence and medical oversight** emerged from the high similarity score between "take care of yourself", "to take my ARV medication", and "to collect my treatment', suggesting that for individuals managing HIV, the concept of proper treatment was tightly bound to the specific actions of medication adherence and access.

- **Health monitoring and professional support** emerged from the high similarity score between “to be followed by my doctor” and “to check my viral load”, suggesting that ongoing medical supervision and regular health monitoring were perceived as two sides of the same coin.

These results allowed us to make comparative insights between and across groups:

- **Gender-specific concerns**: Women’s themes heavily emphasized information-seeking, proactive health management, and the importance of continuous medical support, reflecting a nuanced approach to managing a chronic condition like HIV. In contrast, themes among men, particularly HIV negative, were more varied, reflecting concerns about physical health, social support, and situational vulnerabilities.
- **HIV status influence**: HIV positive participants, regardless of gender, showed a strong focus on proactive health management, treatment adherence and regular monitoring, highlighting the chronic nature of HIV management. In contrast, HIV negative participants showed a broader range of concerns, reflecting preventative and immediate health concerns.
- **Role of social and psychological support**: Both groups highlighted the importance of social and psychological support, but the context differed. For HIV negative men, it was more about motivators or barriers, while for HIV positive participants, it was tied to seeking security and managing vulnerability.

These insights could inform service providers about tailoring offerings to meet the distinct needs of each subgroup, emphasizing the importance of psychological support, clear communication about health impacts, targeted risk reduction, peer-led inductions, and specialized services for situational vulnerabilities.
