# Supplementary material for: Optimising adolescents and young adults’ utilisation of sexual and reproductive health and HIV services in Chad: a sensemaking approach
Source: BMJ Glob Health. 2025 Mar 26;10(3):e017763. doi: 10.1136/bmjgh-2024-017763 (PMC11950941; doi:10.1136/bmjgh-2024-017763)
Supplement: online supplemental file 4 [file bmjgh-10-3-s004.pdf]

S4 File: Summary of results for pile sorting activities regarding no access to care.

When comparing based on HIV status and further stratifying by gender, we observed distinct themes and priorities that reflect their unique concerns, motivations, and needs regarding SRH and HIV services.

For HIV negative male and female participants, below is the similarity matrix:

Themes		1	2	3	4
1	Emotional/psychological	1.0	0.0	0.0	0.6
2	Financial	0.0	1.0	0.5	0.0
3	External barriers	0.0	**0.5**	1.0	0.0
4	Social influence	**0.6**	0.0	0.0	1.0

From this similarity matrix, the following themes were identified:

- **Internal barriers** emerged from the moderate similarity score between “social influence” and “emotional and psychological barriers”, indicating that internal, personal nature of “emotional and psychological barriers” could be closely related to the social influences exerted by family and society, as both could impact an individual’s internal state and decision-making process.
- **Practical barriers** emerged from the moderate similarity score between “financial barriers” and “external barriers”, indicating that they could overlap in their impact on an individual’s ability to access care.

For HIV positive male participants, below is the similarity matrix:

Themes		1	2	3	4	5
1	Healthcare staff issues	1.0	0.0	0.0	0.0	**0.8**
2	External barriers	0.0	1.0	0.0	**0.7**	0.0
3	Emotional barriers	0.0	0.0	1.0	0.0	0.0
4	Accessibility barriers	0.0	**0.7**	0.0	1.0	0.0
5	Interpersonal barriers	**0.8**	0.0	0.0	0.0	1.0

From this similarity matrix, the following themes were identified:

- **Interpersonal dynamics** emerged from the high similarity score between “healthcare staff issues” and “interpersonal barriers”, reflecting the common theme of interactions between healthcare providers and patients. This suggested that issues such as lack of staff knowledge, negligence, and poor reception were all part of the broader of how healthcare staff engaged with patients, impacting the patient experience and willingness to seek care.
- **Logistical challenges to access** emerged from the high similarity score between “external barriers” and “accessibility barriers”, highlighting the logistical obstacles that individuals faced when trying to access healthcare services. This indicated that factors like lack of funding for transport, work preoccupations, and the physical distance to healthcare facilities all contributed to the practical difficulties in accessing healthcare.

For HIV positive female participants, below is the similarity matrix:

Themes		1	2	3	4
1	Financial and logistical barriers	1.0	0.0	0.0	0.5
2	Emotional barriers	0.0	1.0	**0.7**	0.0
3	Social barriers	0.0	**0.7**	1.0	0.0

4	Practical barriers	**0.5**	0.0	0.0	1.0

From this similarity matrix, the following themes were identified:

- **External constraints** emerged from the moderate similarity score between "financial and logistical barriers" and "practical barriers", indicating that external and practical aspects of accessing healthcare, such as cost and time, could sometimes overlap in their impact on individual's decisions.
- **Psychosocial barriers** emerged from the high similarity score between "emotional barriers" and "social barriers" reflecting the internal and psychological nature of these barriers. This suggested that emotional responses such as fear could often be intertwined with social experiences of discrimination and stigmatization.

These results allowed us to make comparative insights between and across groups:

- **Gender and HIV status influence**: The comparison revealed that the impact of various barriers could differ significantly based on both gender and HIV status. For instance, interpersonal dynamics within healthcare settings were particularly salient for HIV positive males, while psychosocial barriers were notably significant for HIV positive females.
- **Internal vs. external barriers**: HIV negative males seemed to be influenced by a combination of internal (emotional and psychological) and external (financial and informational) barriers, whereas for HIV positive participants, the nature of barriers shifted more towards interpersonal dynamics and logistical challenges for males, and external constraints and psychosocial barriers for females.
- **Role of healthcare provider interactions**: For HIV positive males, interactions with healthcare providers emerged as a distinct and critical factor, suggesting the need for interventions focused on improving healthcare provider-patient relationships.

These insights underscored the complexity of healthcare access barriers and the importance of considering individual demographics and health status in developing targeted interventions to improve access to SRH and HIV services.
