# Supplementary material for: Optimising adolescents and young adults’ utilisation of sexual and reproductive health and HIV services in Chad: a sensemaking approach
Source: BMJ Glob Health. 2025 Mar 26;10(3):e017763. doi: 10.1136/bmjgh-2024-017763 (PMC11950941; doi:10.1136/bmjgh-2024-017763)
Supplement: online supplemental file 5 [file bmjgh-10-3-s005.pdf]

S5 File: Examples of coding process.

Initial codes:
Transcript segment 1: Experiences with SRH and HIV care (HIV positive participants)
BE: If I mention SRH care, what would you think of? What does SRH mean to you?
P32: It involves sexually transmitted diseases. Seek to be healthy and protect your partner.
P30: It calls for the fight against diseases such as STIs using preventive or protective measures to avoid illnesses.
P33: It deals with protection against STIs.
P31: When you need to protect your partner from certain diseases such as STIs.
P32: Seeking to put up a barrier or to cure HIV (making ARVs available to patients providing moral support to help them overcome their state of health).
P30: Care actions to stop or reduce the spread of HIV through ARV treatment.

Open coding
P32: It involves **sexually transmitted diseases**. **Seek to be healthy** and **protect your partner**.
P30: It calls for the **fight against diseases such as STIs** using preventive or protective measures to **avoid illnesses**.
P33: It deals with **protection against STIs**.
P31: **When you need to protect your partner from certain diseases such as STIs**.

**Codes**	**Categories**	**Description**
Focus on STIs<br>Preventive measures<br>Illness avoidance<br>Disease combat<br>STI protection focus<br>Barrier creation<br>HIV spread prevention	Disease prevention and health promotion	Youth perceive SRH and HIV care primarily as a means to prevent diseases, particularly STIs and the spread of HIV. The focus is on combatting diseases through strategies that minimize risk and protect health.
Partner protection	Relational health responsibility	A significant theme in youth's responses is the responsibility to protect partners from health risks. This relational aspect of healthcare reflects a broader understanding that health decisions impact not only the individual but also their partner, emphasizing a shared responsibility for disease prevention.
HIV cure effort<br>ARV provision<br>ARV treatment focus	HIV specific care	When discussing HIV care, youth highlighted efforts to cure HIV or manage its spread, particularly through the provision of ARVs. This underscores a specialized approach to HIV care, focusing on both medical treatment and the broader goal of reducing HIV transmission.

Moral support	Supportive care	Beyond physical health measures, youth recognized the importance of moral and emotional support in managing health, particularly for HIV care.
Health maintenance		

Constant comparative approach:
Transcript segment 2: Experiences with SRH and HIV care (HIV positive participants)
BE: What services do you see as being included under SRH and HIV care? Where do you get most of your information?
P35: I am thinking about **hygienic services, health advice, pediatrics, maternity, and gynecology**.
P36: When I think about services for SRH, I think about **antenatal consultation and postnatal consultation**.
P34: **Social media and radio**.
P36: I get my information **from school**.
P37: In the **hospital**.
P34: When you feel weak, need alone, fertility issues.
P35: **The government can provide services in each neighborhood**, the permanence of electricity. Recruit several doctors, provide education, increase technical facilities, create new services.
P36: **Public because the cost is affordable and it's everywhere in the city**.
P34: It's **difficult to access care** because of **travel and doctors' tardiness**.
P37: **It's easy because of the relationships with the doctors**.
P35: **Cases of sexual intercourse or need for specific HIV and SRH care**. **During pregnancies when you are sick**.

Open coding

Codes	Initial coding	Categories
Hygienic services, health advice, pediatrics, and gynecology.	Comprehensive SRH services	Services
The government can provide services in each neighborhood.	Government support	Services
Antenatal consultation and postnatal consultation.	Maternal health services	Services
Public because the cost is affordable and it's everywhere in the city.	Accessibility of public services	Services
Social media and radio	Media as information source	Information sources
It's difficult to access care because of travel and doctors' tardiness	Access barriers	Barriers to care
It's easy because of the relationships with the doctors.	Ease due to relationships	Relationship with healthcare providers
Cases of sexual intercourse or need for specific HIV or SRH care.	Event-driven care usage	Specific instances
During pregnancies when you are sick.	Pregnancy-related care	Specific instances

Constant comparison with previous data

We compared the new codes with those identified from Transcript Segment 1 to refine categories and identify patterns:

- **Comprehensive SRH Services** (P35) and **Maternal Health Services** (P36) can be compared with **Disease Prevention and Health Promotion** from segment 1 to enhance our understanding of what services are deemed necessary.
- **Media Information Source** (P34) and **Access Barriers** (P34) should be compared with **Diverse Information Sources** and **Challenges in Accessing Services** to understand the broader context of information dissemination and access challenges.
- **Event-Driven Care Usage** (P35) and **Pregnancy-Related Care** (P35) relate to the **Usage Motivations** from segment 1, emphasizing the specific events that trigger the use of health services.
- **Ease Due to Relationships** (P37) offers a new dimension to Interaction with Healthcare Provider from segment 1, highlighting how personal relationships can influence the ease of accessing care.

Refining categories and developing themes

**Refined categories**	**Emerging themes**	**Codes**
Service types and needs	Youth see a range of services as part of SRH and HIV care, from disease prevention to comprehensive reproductive services, emphasizing a broad understanding of health needs.	Comprehensive SRH services
		Maternal health services
		Disease prevention and health promotion
Information sources and access challenges	Youth face several barriers to accessing SRH and HIV services, with information sources varying widely from media to personal relationships, impacting their service utilization.	Media as information source
		Diverse information sources
		Access barriers
		Challenges in accessing services
Interactions with healthcare providers	The quality of personal relationships with healthcare providers can significantly ease or complicate youth's access to and use of SRH and HIV services.	Ease due to relationships
		Interaction with healthcare provider
	Specific life events and health concerns, such as sexual activity and pregnancy, are primary drivers behind youth seeking SRH and HIV care.	Event-driven care usage
		Pregnancy-related care
		Usage motivations

By using the constant comparative method, we continually refine our understanding of the data allowing for the development of grounded, nuanced themes that capture the complexities of youth's experiences with SRH and HIV care. This iterative process is central to constructivist grounded theory, helping to ensure that the emerging theory is rooted in participants' perspectives and experiences.
