# Supplementary material for: Optimising adolescents and young adults’ utilisation of sexual and reproductive health and HIV services in Chad: a sensemaking approach
Source: BMJ Glob Health. 2025 Mar 26;10(3):e017763. doi: 10.1136/bmjgh-2024-017763 (PMC11950941; doi:10.1136/bmjgh-2024-017763)
Supplement: online supplemental table 1 [file bmjgh-10-3-s006.pdf]

S1 Table: Stage 1 identified themes for sensemaking

The iterative analysis of responses from AYA revealed that their actions and understanding were deeply rooted in interpreting complex, multifaceted social and personal experiences. This aligned well with the theoretical concept of sensemaking, where individuals construct meaning and guide their behavior through interactions, reflection, and contextual understanding. Therefore, applying a sensemaking lens for the design of subsequent stages was justified and beneficial for deepening the exploration of how adolescents navigate SRH and HIV care.

Key observations from stage 1 data	Data insight	Relevance to sensemaking framework
**Complex and personal interpretation of SRH and HIV care**	Participants relied on peers, cultural centers, and healthcare providers, emphasizing shared experiences and community discussions.	AYA actively interpret and internalize information from their environment, building an understanding of SRH and HIV care, which is central to sensemaking.
**Influence of expectations and prior knowledge**	Engagement and behavior were shaped by participants' expectations and knowledge gaps.	Sensemaking involves using past knowledge and expectations to interpret current situations, reflected in how participants navigated SRH and HIV care.
**Reflections on past experiences**	Experiences with healthcare services, including issues of shame, fear, and trust, influenced future actions.	Adolescents' retrospective sensemaking of past experiences guided their future behavior, showcasing a core aspect of the framework.
**Decision-making and prioritizing choices**	Participants prioritized choices related to safe sex practices, trusted sources of information, and accessing health services based on social pressures and perceived risks.	Decision-making influenced by contextual cues and multiple factors aligns with sensemaking, which involves constructing meaning and guiding actions.
