# Supplementary material for: Optimising adolescents and young adults’ utilisation of sexual and reproductive health and HIV services in Chad: a sensemaking approach
Source: BMJ Glob Health. 2025 Mar 26;10(3):e017763. doi: 10.1136/bmjgh-2024-017763 (PMC11950941; doi:10.1136/bmjgh-2024-017763)
Supplement: online supplemental table 2 [file bmjgh-10-3-s007.pdf]

S2 Table : Example of initial, focused, and theoretical coding.

<table>
<tr><th>Raw data</th><th>Initial coding</th><th>Focused coding</th><th>Refined coding</th><th>Theoretical coding</th></tr>
<tr><td>The patient was very cooperative and accepted her serology but insisted on confidentiality.</td><td>Ensuring patient privacy</td><td rowspan="2">Confidentiality and trust building</td><td rowspan="3">Navigating barriers</td><td rowspan="3">Empowering youth in health decision-making through adaptive sensemaking[*]</td></tr>
<tr><td>Since she was a minor patient who has just learned of her HIV status, I had to take more time with her to explain what to do.</td><td>Building trust and availability</td></tr>
<tr><td>Make patients laugh and make the atmosphere pleasant so that the patient feels safe and breaks down barriers.</td><td>Providing emotional support and encouragement</td><td>Personal connection</td></tr>
</table>

[*]This is just an example; however, the theoretical code presented is the final code obtained when considering other focused codes, including collective sensemaking, and adolescent-centered design.
