# Supplementary material for: Optimising adolescents and young adults’ utilisation of sexual and reproductive health and HIV services in Chad: a sensemaking approach
Source: BMJ Glob Health. 2025 Mar 26;10(3):e017763. doi: 10.1136/bmjgh-2024-017763 (PMC11950941; doi:10.1136/bmjgh-2024-017763)
Supplement: online supplemental table 3 [file bmjgh-10-3-s008.pdf]

S3 Table : Additional details on the coding tree.

Number codes	Initial codes	Focused coding
22	Feeling unwell; Risks associated with unprotected sex; Proactive health seeking; Immediate concern and subsequent relief; Managing HIV condition; Engaging with ART; Seeking moral support; Prevention after risky behaviors; Reflective process and commitment to safer practices; Internal dialogues and reflections; Ongoing consideration and challenges in accessing care; Symptom recognition; Influence of past experiences; Health information seeking; Emotional response to health status; Adaptation to health challenges; Navigating health systems; Perceived severity of health issues; Financial constraints on health decisions; Impact of health knowledge on behavior; Anticipation of health risks; Commitment to health maintenance.	Individual sensemaking
6	Consulting elders; Available information; Advice from healthcare workers; Discussing with family members in healthcare; Reliance on family for healthcare decisions; Utilization of community and media resources.	Process of seeking information
8	Advice from elders and credibility; Parental involvement and support; Family members in healthcare as influencers; Building trust with healthcare workers and community actors; Community actors offering hope and understanding; Pharmacists as accessible healthcare advisors; Emotional support and	Interpersonal influence

	reassurance through interpersonal interactions; Trust and accessibility in seeking advice.	
17	Trust in healthcare providers; Confidentiality in care; Privacy in care; Supportive and non-judgmental environment; Physical and emotional safety; Impact of healthcare provider's demeanor; Role of social clubs in building trust; High standards of professionalism and ethics; Navigating healthcare with trust and safety; Valuing expertise and credibility; Creating a safe haven for health discussions; Ensuring accessibility and approachability of healthcare services; Cultural competence and understanding; Facilitating open communication; Influence of societal perceptions; Stigma-free perceptions; Responsive and patient-centered care.	Trust and safety
10	Financial constraints limiting access; Psychological barriers to care; Geographical proximity and accessibility of care; Professionalism and attitude of healthcare staff; Quality of care provided; Effective communication by healthcare providers; Stigma-free perceptions; Responsive and patient-centered care; Updated knowledge; Empathetic and competent care.	Challenges in HIV care
5	Physical proximity; Youth-specific services; Membership and availability; Affordability; Accessibility.	Customized accessibility
8	Adaptability of healthcare providers; Sensitivity to adolescent needs; Engagement with youth; Family	Responsiveness of care

	involvement in care; Psychosocial understanding in care; Empathetic and supportive care environment; Accessibility and follow-up in care; Holistic and integrated care approach.	
4	Evidence-based interventions; Specialized training; Multifaceted considerations in care; Developmental considerations in care.	Clinical effectiveness
8	Individualized approach in care; Flexible and informal interaction settings; Personalizing care pathways and communication; Culturally and contextually appropriate interventions; Peer-to-peer engagement in health education; Comprehensive approach to adolescent life; Understanding and integrating family dynamics; Adapting to health literacy levels.	Tailored service delivery
